# OD39 Evaluation Of Sustainability And Environmental Impact Measures In Health Technology Assessment Of Medical Technologies In Europe: Starting An Important Journey

**DOI:** 10.1017/S0266462324001685

**Published:** 2025-01-07

**Authors:** María Álvarez Orozco, Julen Monje, Natalie Hallas, Ilona Vogt-Humberg, Francesca Borghetti, Constance Mauratille, Liesl Strachan

## Abstract

**Introduction:**

Reducing environmental impact and ensuring sustainable practices are important to the medical technology (MedTech) industry. However, it is unclear how these factors will be formally addressed by health technology assessment (HTA). The objective of this research was to understand what sustainability measures are required and included in HTA reports on medical technologies in five European countries (France, England, Germany, Italy, and Spain).

**Methods:**

HTA guidelines, framework papers, and published HTA reports for devices were searched from January 2022 to November 2023 for inclusion of environmental aspects. Search terms included environmental sustainability, carbon footprint, greenhouse gas, and waste generation. Data were extracted into standardized templates and analyzed both quantitively and qualitatively for the inclusion of sustainability and environmental impact measures.

**Results:**

HTA guidance on inclusion of sustainability and environmental measures is lacking. While some countries request the inclusion of these aspects in the HTA process, there is little detail on evidence requirements and how it will be evaluated. Of the over 450 HTA reports examined, less than 10 percent included environmental aspects, with most sustainability benefit claims made related to the reduction in waste or less resources used. Very few claims were supported by published evidence. This resulted in the exclusion of potential environmental benefits in final HTA recommendations. Costs associated with environmental impact of medical technologies were rarely assessed.

**Conclusions:**

Current inclusion and evaluation of sustainability measures in MedTech HTA in Europe is nascent. While countries like England [National Institute for Health and Care Excellence (NICE)] and France [Haute Autorité de santé (HAS)] have very recently made public statements regarding the importance of environmental impact, there is a growing need for detailed guidance on environmental evidence requirements and how these will be incorporated into the evaluation and decision-making processes.

